# Dietary Quality among Tehranian Adults in Relation to Lipid Profile: Findings from the Tehran Lipid and Glucose Study

**DOI:** 10.3329/jhpn.v31i1.14747

**Published:** 2013-03

**Authors:** Golaleh Asghari, Parvin Mirmiran, Firoozeh Hosseni-Esfahani, Pantea Nazeri, Mahya Mehran, Fereidoun Azizi

**Affiliations:** Obesity Research Center, Nutrition and Endocrine Research Center, Research Institute for Endocrine Sciences, Shahid Beheshti University of Medical Sciences, Tehran, Iran

**Keywords:** Diet quality index-international, Healthy eating index-2005, Lipid profile, Mediterranean diet scale

## Abstract

The prevalence of dyslipidaemia has been increasing in developing countries that are undergoing nutrition transition. However, the association of diet quality and lipid profile has not been well-understood in these countries. The aim of the current study was to compare the ability of three diet quality indices—the Mediterranean diet scale (MDS), healthy eating index-2005 (HEI-2005), and diet quality index-international (DQI-I) in relation to changes in lipid profile between baseline and 6.7 years of follow-up. Baseline data from two 24-hour dietary recalls provided by 469 adults with mean age of 38.7±12.3 years, who were participants of the Tehran Lipid and Glucose Study, were analyzed to describe dietary intakes. Data on anthropometry, sociodemography, physical activity, and other lifestyle variables were recorded, and a comparison of baseline and follow-up data revealed changes in the concentrations of total cholesterol (TC), triglyceride (TG), high-density lipoprotein cholesterol (HDL-C), and low-density lipoprotein cholesterol (LDL-C). A general linear model was used in assessing changes in lipid profile depending on adherence to diet quality indices at baseline, after adjustment for age, smoking status, waist-circumference, body mass index, physical activity, and energy intake. A significant interaction was observed between scores and sex, and upon stratification, males in the highest quartile score of the HEI-2005 had significantly lower TG changes than those in the lowest quartile, after adjusting for confounders (-8.8 vs 2.9, p=0.038). No association was apparent in females (-0.2 vs 11.2, p=0.297). In addition, a positive association was found between DQI-I and HDL-C (Q1=0.6 vs Q4=-2.0, p=0.044) in males. In both sexes, all these indices were weakly associated with TC while none was associated with LDL-C. It is concluded: adherence to the HEI-2005 recommendations was weakly associated with reduced TG concentration in an urban Iranian adult population. The MDS and DQI-I were not related to change in lipid profile.

## INTRODUCTION

In recent years, both posteriori and the priori dietary patterns have been used in the evaluation of diet and chronic diseases (1,2). The posteriori dietary pattern is based on statistical modelling of dietary data, and the priori dietary pattern is the theoretical dietary pattern known as diet quality indices. The healthy eating index-2005 (HEI-2005) and the diet quality index-international (DQI-I) are two theoretical dietary patterns developed as measurement of adherence to dietary guidelines and the food guide pyramid (3,4); however, the Mediterranean dietary scale (MDS) was developed as a measurement of the Mediterranean dietary pattern (5). The MDS was widely based on low rates of chronic diseases and mortality (6,7). The HEI-2005 has been constructed in revision of original HEI, following the publication of the 2005 Dietary Guidelines; it has put more emphasis on important aspects of diet quality, such as whole grains, various types of vegetables, specific types of fat, and the introduction of the new concept of “discretionary calories” (4). The DQI-I assesses diet quality across countries at different stages of the nutrition transition and provides information not only on chronic diseases but also on problems of undernutrition (3). When diet quality scores show correlations with nutrient intakes and serum biomarkers and are tested with health outcome or disease risk factors, they are validated (8) as found for HEI and MDS (5,9,10).

Dyslipidaemia, a major risk factor of chronic disease, has been shown to have a high prevalence worldwide, specifically in developing countries (11). According to the third national surveillance of risk factors of non-communicable diseases conducted in 2007, the prevalence of hypertriglyceridaemia and hypercholesterolaemia were 36.4% and 42.9% respectively in Iranian population (12). Healthy dietary patterns may improve the risk of developing dyslipidaemia (13). Shah *et al.* reported that the HEI-2005 was a negative predictor of low-density lipoprotein cholesterol (LDL-C) and total cholesterol (TC) and a positive predictor of high-density lipoprotein cholesterol (HDL-C) among American women postpartum, from 2004 through 2007 (14). Furthermore, higher adherence to dietary guidelines was related to decrease in the risk of low HDL-C cross-sectionally, using the dietary guidelines for Americans index (DGAI) (15) in Tehran Lipid and Glucose Study subjects whereas there were no significant correlations between HEI and lipid profiles in NHANES III (9). Substantial health benefits of dietary scores in relation to glucose profile (16,17), adiponectin (18,19), inflammation (20), and antioxidant profile (9) have been reported previously. Regardless of the fact that there is little evidence to link favourable dietary scores to improved lipid profile status, there are no quantitative dietary guidelines in Iran to evaluate healthy dietary patterns and their relationship with risk factors. Therefore, an applicable and feasible dietary score in a specific population to predict risk factors in a prospective framework may help policy-makers design and establish suitable interventional strategies and programmes.

The aim of the current study was to compare the ability of three diet quality indices―HEI-2005, MDS, and DQI-I―to predict changes in lipid profile during 6.7 years of follow-up in Tehranian adults.

## MATERIALS AND METHODS

### Study design and population

The present study was conducted within the framework of the Tehran Lipid and Glucose Study (TLGS), an ongoing study being performed in district no.13 of Tehran, the capital of Iran, to detect and prevent risk factors of non-communicable diseases (21,22).

Following the enrollment of 15,005 participants aged ≥3 years by multistage cluster random-sampling method from three medical health centres at baseline (1999-2001), the individuals were invited every three years thereafter to update their demographic, lifestyle, medical and dietary information and biochemical measurements. Of 15,005 participants 1,476 (818 subjects aged ≥19 years) were randomly selected for dietary intake assessment. Of these participants, 517 subjects had valid dietary data at baseline and completed the third phase (2006-2008), with the median follow-up of 6.7 years (response rate 67.0%). After excluding pregnant and lactating women (n=14), participants with special diets and those who used medication for dyslipidaemia (n=34), over- and under-reporters (n=5), and those with incomplete data on TC (n=13), TG (n=13), HDL-C (n=14), and LDL-C (n=58), the final sample-size was 451 for TC and TG, 450 for HDL-C, and 406 for LDL-C.

This study was approved by the ethics committee of the Research Institute for Endocrine Sciences, Shahid Beheshti University of Medical Sciences, and informed written consent was obtained from each subject.

### Assessment of dietary intake

Dietary information was ascertained at baseline and was assessed using a 24-hour dietary recall (24-hDR) on two randomly-selected non-consecutive days. There was at least a 10-day interval between collecting the 24-hDRs; the two recall days were among weekdays when participants had usual dietary intakes. Expert nutrition interviewers, with at least 5 years of experience in the nationwide food consumption survey project (23), were assigned to each participant to collect information on dietary intake on the two recall days. To determine the volume of the household measures, the first 24-hDR interview was performed at the subject's home, and the second 24-hDR was completed at the TLGS nutrition unit by the same interviewer. The 24-hDR is based on actual intake and may be used in estimating absolute rather than relative intake (24); it is, however, susceptible to recall bias, both for identification of foods eaten and for quantification of portion-sizes. This type of error is reduced through gathering dietary data by highly-trained interviewers. Since recalling 2 days cannot cover all day-to-day variations in dietary intake, the use of non-consecutive days enhances the coverage (25). For each food item, a portion-size was specified using USDA serving-sizes (e.g. bread-1 slice; apple-1 medium; dairy-1 cup) whenever possible. If this was not possible, household measures (e.g. beans-1 tablespoon; chicken meat-1 leg, breast, or wing; rice-1 large, medium, or small plate) were chosen. The portion-size of consumed foods was converted to grammes, and the amounts of nutrient and energy contents were calculated according to Nutritionist III (N3) (version 3.0; N-squared Computing, Salem, OR, USA) modified according to the Iranian Food Composition Table (26). The mean of two dietary recalls were applied for data analysis. Over- or under-reporters were defined as participants who had ±3 SD of the proportion of energy intake to estimated energy requirement ratio.

### Construction of modified diet quality scores

The DQI-I, defined by Kim *et al.* (3), was constructed based on four aspects of a healthy diet, including variety, adequacy, moderation, and balance. Variety was assessed by two components, including ‘between-food groups’ (0-15 points) and ‘within-protein sources group’ (0-5 points) on a category scale. The maximum score for the within-protein group was achieved by intakes of half serving from protein sources. Adequacy evaluates the vegetable, fruit and grain group, fibre, protein, iron, calcium and vitamin C intakes (forty scores). The scoring of food groups and fibre was based on three energy levels, including 1,700, 2,200, and 2,700 kcal. Moderation is based on five components, including total fat, saturated fat, cholesterol, sodium, and empty calorie foods on a category scale (thirty scores). Not having reliable data on sodium intake, we scored sodium according to the distribution of intake by study subjects. Individuals who consumed sodium over the 85^th^ and less than the 15^th^ percentile had 0 and 6 points respectively. The balance component examines the balance in macronutrient distribution of diet and fatty acid ratio on a category scale (10 points). The ratio of carbohydrate:protein:fat assigns corresponding points; a ratio of 55~65:10~15:15~25 gets six points, one of 52~68:9~16:13~27 gets 4 points, a ratio of 50~70:8~17:12~30 gets 2 points, and others get zero point. Regarding the balance of fatty acid ratio in terms of polyunsaturated fatty acid/saturated fatty acid and monounsaturated fatty acid/saturated fatty acid, the participant gets 4 points if the ratios for both are 1~1.5, gets 2 points if the ratios for both are 0.8~1.7, and gets zero point if the ratios are otherwise. The total possible score was 100. A higher score indicated a better quality of diet (Appendix 1).

The HEI-2005 (4) was proposed by Guenther *et al*. following the release of the MyPyramid and dietary guidelines in 2005. The index evaluates two major concepts (adequacy and moderation) of the usual diet through 12 components, including total and whole fruit and grains; total, dark-green and orange vegetables and legumes; milk; meat and beans; oils; energy from saturated fat, solid fats and added sugars. For the current study, we applied two modifications to the original index. Since we did not have reliable data on alcohol intake, we decided on a priori to eliminate the alcoholic beverages component; as there were no valid data on table salt intake, the sodium content of foods was derived in grammes. The deciles of sodium content of foods were calculated; 10 points were assigned to the lowest decile (245.6 g) and 0 to the highest decile (1232.2 g). Legumes are counted as the vegetables group, only after the meat and beans component criteria have been met. The discretionary calories allowance is the total calories from solid fats and added sugars (SoFAAS component). Solid fat calories were defined as fat (in grammes) consumed from butter, margarine, hydrogenated vegetable oil, and cream. In addition, total excess fat in grammes beyond what would be consumed if only the lowest forms of fats were eaten, was considered as solid fat calories. Calories from added sugars were obtained from sugar added to tea and coffee, chocolate milk, ice cream, peanut butter, sweet rolls, cakes, cookies, baklava, pastries, doughnuts, crackers, ready-to-eat cereals, fruit juice, canned fruit, nectars, mayonnaise, syrup, honey, jelly, jams, candy, caramel, chocolate, halvah, toffee, and soft drinks. The intakes of foods and nutrients were assessed on the basis of the energy intake density. The energy intake of each individual was calculated, and the intakes of foods and nutrients were presented per 1,000 kcal energy intake, except for saturated fat and SoFAAS. The total score of HEI-2005 index ranged from 0 to 100, higher scores indicating a better diet quality (Appendix 2).

The traditional MDS was created to measure the level of adherence to the Mediterranean diet (5). It included nine components: vegetables, legumes, fruits and nuts, cereals, fish, meat, dairy products, alcohol, and the ratio of monounsaturated fatty acid (MUFA) to saturated fatty acid (SFA). Sex-specific median intakes of food groups were calculated. In the current study, the original MDS was modified as follows:

The fruits and nuts component was divided into two groups, the alcohol component was removed, the ratio of red to white meat was considered a negative point, whole grains and refined grains were separated, and polyunsaturated fatty acid (PUFA) was substituted for MUFA intake. The final components of the modified MDS were: (i) fish, (ii) fruits, (iii) legumes, (iv) nuts, (v) ratio of PUFA to SFA, (vi) vegetables, (vii) whole grains, (viii) refined grains, (ix) dairy products, and (x) ratio of red and processed meats to white meat. A value of 1 was attributed to fish, fruits, legumes, nuts, ratio of PUFA to SFA, vegetables, and whole grains, if the intake was higher than the median; and a value of 1 was attributed to refined grains, dairy products, and the ratio of red and processed meats to white meat if the intake was lower than the median; otherwise, 0 was attributed to each component. Thus, the total score ranged from 0 (lowest adherence) to 10 (highest adherence) (Appendix 3).

### Assessment of other variables

All information regarding age, sex, smoking status, physical activity, and anthropometric measures was recorded at baseline, using validated questionnaire (21). The smoking status was assessed as daily or occasional smoking and non-smoking. Data on participant^’^s physical activity, which have been reported earlier, were obtained using the Lipid Research Clinic (LRC) questionnaire (27). This questionnaire is a simple and comprehensible measure, including four questions; no special education is needed to complete this questionnaire. Subjects were classified as having low, moderate and high levels of physical activity based on their oral responses to the questionnaire. Anthropometric measurements, including weight, height, and waist-circumference (WC), were measured by trained technicians. Weight was measured while subjects were minimally clothed without shoes, using digital scales and recorded to the nearest 100 g (Seca 707, Seca Corp., range 0.1-150 kg, Hamburg). Height was measured using a tapemeter (Model 208 Portable Body Meter Measuring Device; Seca) while participants were in standing position without shoes with their heads in the Frankfort horizontal plane and recorded to the nearest 0.5 cm. BMI was calculated as weight in kg divided by squared height in metres (kg/m^2^). WC was measured at the level of the umbilicus over light clothing, using unstretched tapemeter, without any pressure to body surface and was recorded to the nearest 0.1 cm (21).

### Biochemical measurements

From each study participant, a blood sample was drawn between 7:00 and 9:00 am, after 12-14 hours of fasting, at baseline, and after 6.7 years of follow-up. All blood analyses were done at the laboratory for TLGS. The analysis of samples was performed using selectra 2 auto-analyzer (Vital Scientific, Spankeren, Netherlands). For measurement of triglycerides, we used an enzymatic calorimetric method, with glycerol phosphate oxidase. Inter- and intra-assay coefficients of variation (CV) for TG were 0.6 and 1.6% respectively. TC was assessed with cholesterol esterase and cholesterol oxidase by using enzymatic colorimetric method. After precipitation of the apolipoprotein β with posphotungstic acid, the HDL-C was measured. Inter- and intra-assay CV for both TC and HDL-C were 0.5 and 2% respectively. LDL-C was calculated from the serum TC, TG and HDL-C concentrations expressed in mg/dL, using the Friedewald formula (28). These analyses were performed using commercial kits (Pars Azmoon Inc., Tehran, Iran). The biochemical measurement methods, applied at baseline and after 6.7 years of follow-up, were the same.

### Statistical analysis

Descriptive statistics of the baseline demographic and healthy diet characteristics of the TLGS participants were described using mean and standard deviation (SD), after being tested for normal distribution for quantitative variables and percentages for qualitative variables, and were tested for a trend across levels of adherence to diet quality indices by linear regression for continuous variables and Cochran-Armitage test for categorical variables (29). The continuous measures of the scores were categorized into quartiles for further analyses. There were significant interactions by sex on the association of indices and lipid profile. The outcome variables were changes that occurred in the concentrations of TC, TG, HDL-C, and LDL-C between baseline and end of the follow-up. Since distribution of TG and HDL-C changes were not normal, a logarithmic transformation of these was used. We used a general linear regression model to assess the predicted probability of MDS, HEI-2005, and DQI-I for outcome variables. Our first model (Model 1) included diet quality indices as independent variables and changes in lipid profile as dependent variables. Age (in years) was added to Model 1 to construct Model 2. Smoking status (reference: no), physical activity (reference: low), BMI (kg/m^2^), WC (cm), and energy intake (kcal) were added to Model 2 to construct Model 3. As the results for these 3 models were relatively similar, Model 3 was reported. Statistical Package for Social Science (SPSS Inc., Chicago IL, USA, version 9.05) was used for all statistical analyses.

## RESULTS

Baseline characteristics of the participants across quartiles of diet quality indices are shown in Table 1. More participants with the highest scores of HEI-2005 were females, and less participants were current smokers. Those with the highest levels of adherence to MDS and DQI-I had high levels of physical activity. The baseline prevalence of TG ≥150 mg/dL, TC ≥200 mg/dL, HDL-C <40 mg/dL, LDL-C ≥130 mg/dL, abdominal obesity, and BMI ≥25 kg/m^2^ was not significant across quartiles of any index.

Compared to the low adherers, those with higher adherence to HEI-2005 consumed less energy. Participants with the highest scores of HEI-2005 and DQI-I tended to have higher intakes of carbohydrate; however, those with the highest scores of MDS tended to have lower intakes of carbohydrate. Intakes of fibre increased across quartiles of MDS and DQI-I whereas that of total fat and saturated fat decreased across quartiles of HEI-2005 and DQI-I. Participants with the highest scores of MDS and HEI-2005 tended to have higher intakes of MUFA and PUFA; however, those with the highest scores of DQI-I tended to have lower intakes of MUFA and PUFA (Table 2). Subjects with higher adherence to HEI-2005 and DQI-I had lower intakes of sweets, and those with higher adherence to MDS and DQI-I had higher intakes of whole grains.

As shown in the figure, DQI-I was negatively associated with HDL-C change (p=0.044) in males, after adjustment for confounders. Subjects in the last quartile of MDS were about 75% and 82% less likely than those in the first quartile to have decrement in HDL-C in males and females respectively; although not significant, a similar trend was seen for HEI-2005 in females (80%). An inverse association between adherence to the HEI-2005 and the predicted probability of TG change in males was observed (p=0.038), after adjusting for confounding variables. The highest quartile of adherence to the HEI-2005 exhibited the highest TG change (-20.0 mg/dL) compared to those in the lowest quartile (11.2 mg/dL). A non-significant, negative linear trend was seen, after adjustment for confounding variables between all indices and changes in TG concentration, both in males and females. No significant relationship was seen between changes in the scores for MDS, HEI-2005, and DQI-I and changes in TC; however, the relationship showed a negative linear trend in males. No significant relationship was found between diet quality scores and alterations in LDL-C concentrations.

The β coefficients of the relationship between components and changes in TG concentration were −0.17, −0.19, −0.18, and −0.24 for whole fruit score in HEI-2005, legumes score in MDS, cholesterol and sodium score in DQI-I respectively in males and were −0.13 and 0.14 for total vegetables score in HEI-2005 and fruit score in DQI-I respectively in females (p<0·05). In the linear regression model, one unit increment in the vegetable scores in MDS and protein scores in DQI-I were associated with −0.16 and −0.15 mg/dL TC changes in males, and dairy and oils in HEI-2005 were associated with −0.16 and 0.17 mg/dL TC changes in females (p<0·05) respectively. Multivariable-adjusted associations between the indices component score and LDL-C change were 0.19 for both whole fruit and SFA component scores in HEI-2005 among males and 0.16 and 0.17 for whole grain and oil component scores in HEI-2005 among females (p<0·05) respectively (data not shown).

## DISCUSSION

In the current study, we evaluated the ability of three diet quality indices, including MDS, HEI-2005, and DQI-I in predicting lipid profile changes after 6.7 years of follow-up in Tehranian population. Comparison of these indices over two time-points indicated that increasing HEI-2005 scores in males was parallel with higher decrements in TG concentration; also, an inverse association between DQI-I and changes in HDL-C concentration was observed in males.

The development of the dietary guidelines requires evidence- and country-based approaches. In the former, the primary source is derived from the results of epidemiologic studies with the high priority being given to prospective studies (30). In the latter, food patterns do not show the same predictive value due to differences in food-usage patterns among the population (31). Hence, due to the limited number of longitudinal studies on diet quality indices and chronic disease risk factors (17,31,32), the current study provides useful information in the establishment of more accurate dietary guidelines in Iran.

Although many studies have evaluated HEI and its different versions, including Alternate HEI (AHEI), HEI-05, and HEI-2005, only a few studies have evaluated the association between HEI-2005 and lipid profiles (14). The present study found a negative association between HEI-2005 and TG in males, which may be attributable to its fruit and vegetable components. This finding was confirmed by previous studies showing the highest association between HEI-2005 and nutrients relating to fruits and vegetables (9). In addition, the associations between HEI-2005 and TG and HDL-C may be attributable to the favourable effects of other dietary factors, such as energy intake and the type and amount of fat intake as documented in our study.

**T1able 1.** Baseline characteristics of the participants across quartiles (Q) of diet quality indices: Tehran Lipid and Glucose Study (n=469) in 1999-2001

Characteristics	MDS				HEI-2005				DQI-I			
	Q1	Q2	Q3	Q4	Q1	Q2	Q3	Q4	Q1	Q2	Q3	Q4
	(≤3)	(4)	(5)	(≥6)	(<50.7)	(50.7-56.7)	(56.8-62.3)	(≥62.4)	(≤60.0)	(60.1-65.1)	(65.2-69.9)	(≥70.0)
Age	37 (2)	39 (12)	39 (12)	38 (12)	36 (10)	37 (12)	41 (13)	39 (12)^†^	38 (11)	36 (12)	39 (12)	40 (12)
Sex (% male)	43.0	49.6	44.8	46.5	45.9	57.5	46.4	33.3^†^	53.6	47.7	39.8	42.3^†^
Educational level												
< Diploma	76.0	71.5	70.1	82.8	72.1	77.0	70.5	81.1	76.7	71.2	70.8	82.0
Diploma	13.4	19.3	16.1	10.1	18.9	11.5	17.9	10.8	17.0	13.5	19.5	9.0
> Diploma	10.6	9.2	13.8	7.1	9.0	11.5	11.6	8.1	6.3	15.3	9.7	9.0
Current smoker (%)	7.4	10.7	7.4	9.9	15.2	6.7	6.7	5.7^†^	13.1	6.8	6.7	8.7
Physical activity (%)												
Severe	18.7	25.9	27.1	34.7^†^	19.4	26.9	27.0	29.7	19.1	22.9	23.2	38.3^†^
Moderate	10.8	14.7	14.1	14.3	12.0	17.6	14.4	9.0	7.3	20.2	10.7	15.0
Low	70.5	59.5	58.8	51.0^†^	68.5	55.6	58.6	61.3	73.6	56.9	66.1	46.7^†^
TGs ≥150 mg/dL (%)	37.9	40.2	40.7	38.4	34.3	38.1	41.4	42.7	38.5	37.6	38.1	42.3
TC ≥200 mg/dL (%)	46.4	40.2	45.3	50.5	38.9	46.9	47.7	48.2	45.9	41.3	49.6	45.0
HDL-C <40 mg/dL (%)	45.0	45.3	51.2	40.4	50.0	50.4	39.6	40.9	45.9	45.9	42.5	46.8
LDL-C ≥130 mg/dL (%)	42.9	42.6	45.8	45.8	41.3	45.0	49.1	40.6	44.8	42.3	46.4	42.6
Abdominal obesity^*^ (%)	33.1	39.8	36.8	37.1	31.8	42.5	32.1	39.4	33.6	33.3	40.7	38.2
BMI ≥ 25 kg/m^2^ (%)	54.2	54.2	60.9	60.8	55.5	61.9	52.7	57.8	50.9	60.4	55.8	60.9

^*^Abdominal obesity was defined as waist-circumference ≥89 and 91 cm for males and females respectively;

^†^p for trend <0.05, using Armitage chi-square for categorical variables and linear regression for continuous variables; no confounders were taken into account; DQI-I=Diet quality index-international; HDL-C=High-density lipoprotein cholesterol; HEI-2005=Healthy eating index-2005; LDL-C=Low-density lipoprotein cholesterol; MDS=Mediterranean diet scale; TC=Total cholesterol; TG=Triglyceride; Data are mean (standard deviation) for continuous variables and percentage for categorical variables

**T1able 2.** Baseline dietary intakes by the participants across quartiles (Q) of diet quality indices: Tehran Lipid and Glucose Study (n=469) in 1999-2001

Intake	MDS		p for trend	HEI-2005		p for trend	DQI-I		p for trend
	Q1 (≤3)	Q4 (≥6)		Q1 (≤50.7)	Q4 (≥62.4)		Q1 (≤60.0)	Q4 (≥70.0)	
Energy (kcal)	2440 (715)	2476 (798)	0.579	2639 (754)	2019 (517)	<0.001	2364 (667)	2371 (725)	0.954
CHO (% energy intake)	59.7 (7.2)	57.8 (7.0)	0.035	56.6 (5.9)	59.7 (7.3)	<0.001	55.1 (6.3)	63.6 (5.3)	<0.001
Sweets (g/d)	49.54 (26.86)	55.58 (43.98)	0.082	75.75 (45.12)	38.74 (22.61)	<0.001	55.64 (32.76)	43.80 (24.65)	0.002
Whole grain (g/d)	10.91 (11.9)	14.31 (10.2)	0.043	11.81 (12.09)	12.36 (11.43)	0.587	10.38 (10.92)	17.31 (13.24)	<0.001
Protein (% energy intake)	11.0 (1.6)	11.7 (2.2)	0.003	10.3 (1.8)	12.2 (2.0)	<0.001	10.6 (1.9)	12.0 (1.9)	<0.001
Fat (% energy intake)	29.3 (7.7)	30.6 (7.5)	0.206	33.1 (6.1)	28.2 (7.6)	<0.001	34.3 (6.4)	24.4 (5.3)	<0.001
Fibre (g/d)	7.2 (3.2)	8.8 (2.8)	0.002	7.2 (3.4)	8.0 (3.2)	0.112	6.1 (2.0)	10.2 (7.7)	<0.001
SFA (% energy intake)	5.75 (7.32)	15.76 (8.56)	0.958	20.47 (8.43)	10.74 (4.54)	<0.001	18.12 (8.23)	11.34 (6.14)	<0.001
MUFA (% energy intake)	6.30 (5.35)	9.11 (6.45)	0.002	7.83 (6.02)	7.81 (6.4)	0.642	9.24 (7.46)	5.75 (4.54)	<0.001
PUFA (% energy intake)	2.18 (3.32)	5.49 (4.49)	<0.001	2.73 (2.5)	5.56 (6.07)	<0.001	4.19 (5.39)	3.14 (3.12)	0.024

CHO=Carbohydrate;

d=Day; DQI-I=Diet quality index-international;

HEI-2005=Healthy eating index-2005;

MDS=Mediterranean diet scale;

MUFA=Monounsaturated fatty acid;

PUFA=Polyunsaturated fatty acid;

SFA=Saturated fatty acid;

Data are mean (standard deviation)

Increased HDL-C concentration plays an important role in CVD prevalence, with reports that increasing one mg/dL of HDL-C concentration will result in 2-3% decrease in CVD (33). In our study, a non-significant positive association was observed between the MDS and HDL-C concentrations. Previous cross-sectional and cohort studies have also shown positive associations between MDS and HDL-C (34,35). A recent meta-analysis by Kastorini *et al.* revealed that the considerable effect of Mediterranean dietary pattern was on HDL-C concentration (36). Babio *et al.* reported that the highest adherence to the Mediterranean diet resulted in a 47% lower prevalence of low HDL-C compared to those with the lowest adherence. After adjusting for BMI, the association was not significant (34). In the SUN (Seguimiento University of Navarra) cohort of Spanish subjects, a marginally-significant HDL-C difference was found between participants with high and low levels of adherence to Mediterranean dietary pattern (35) compared to an approximately 80% non-significant difference in our study, which can be explained by elimination of the alcohol and olive oil, and also adding PUFA/SFA ratio to adapt to Iranian food pattern, which may affect the performance of the index. Moreover, 73%, 60%, and 92% of participants did not consume nuts, whole grains, and fish components respectively. Hence, we cannot apply the current version of this score in our population. In addition, we found a positive non-significant association between HEI-2005 and HDL-C as seen in a recent cross-sectional study on postpartum women (14), in contrast to the negative association Kant *et al.* reported between HEI and HDL-C (10), which was also found in another study evaluating the association of adherence to dietary guidelines with HDL-C (32). It seems that higher scores of the HEI-2005 reflect individuals with high intakes of whole fruit, dark vegetables, whole grains, nuts, and low levels of saturated fat; therefore, its ability to predict lipid profile was expected (14). The DQI-I revealed a significant negative and a non-significant association in males and females respectively. Regarding the studied indices, MDS and HEI-2005 were stronger predictors of HDL-C compared to DQI-I; differences may be attributable to the performance of each index.

**Figure:**
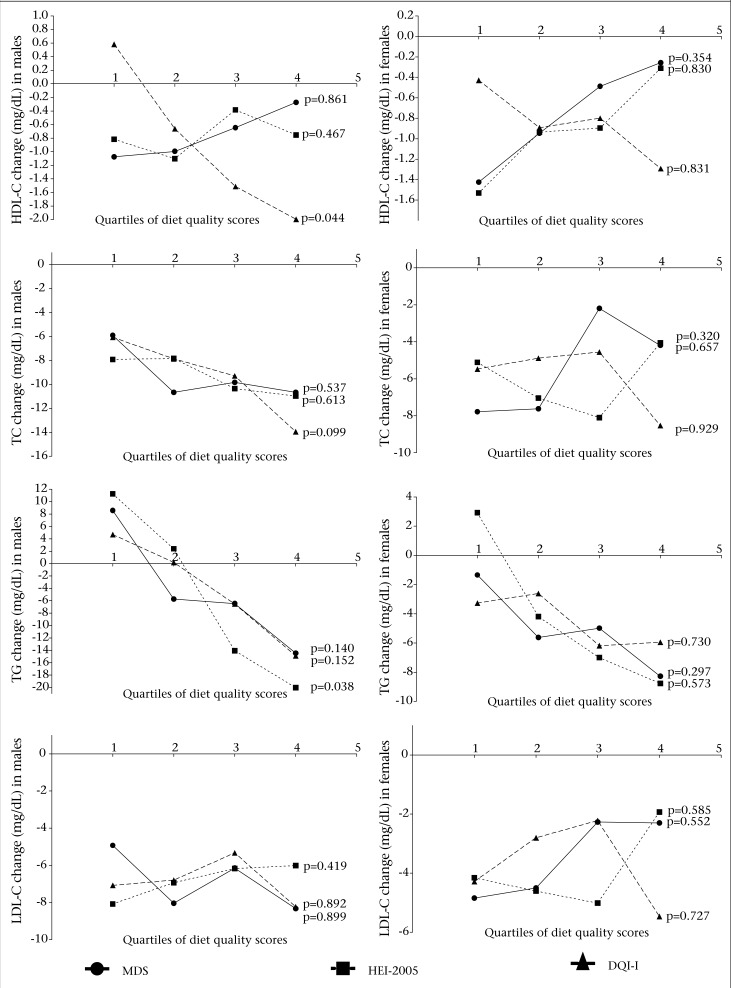
Change in high-density lipoprotein cholesterol (HDL-C), triglycerides (TG), total cholesterol (TC), and low-density lipoprotein cholesterol (LDL-C) by quartiles of Mediterranean diet scale (MDS), healthy eating index-2005 (HEI-2005), and diet quality index-international (DQI-I) adjusted for age, body mass index, waist-circumference, physical activity, smoking (current or no smoker), and energy intakes in males and females, using general linear model regression

### Strengths and limitations

The strengths of the present study are its prospective nature, the long duration of follow-up, and its reliance on a sample of the general population. However, the findings of the current study have some limitations. First, as we used just two dietary recalls, measurement errors were inevitable, and this might lead to underestimation of associations. Although we cannot entirely rule out residual confounding due to erroneous measurement of important covariates, including physical activity, it is unlikely that errors in measuring the covariates would be so extreme to produce the observed associations. As cardio-metabolic risk factors, such as lipid profile concentration, are affected by dietary intakes in the recent past, alteration in dietary pattern during the study period is another limitation that must be mentioned. Furthermore, some potential limitations regarding these indices, are anticipated: first, according to the scoring systems of the HEI-2005 and DQI-I, the indices do not show the extent to which a person deviates from the recommended values; second, none of the diet quality indices considered the recommended energy intake, and negative scores were not allocated to energy intakes over the recommended values. Another possible limitation of indices in our study is the range of indices (HEI-2005 and DQI-I), which was narrow, indicating that scores in the top and bottom quartiles are not far from each other and, consequently, cannot clearly distinguish differences in dietary patterns, which could confound the association of diet and outcome. In addition, subjects with similar scores do not always show similar associations with the outcome (dyslipidaemia in the present study) because the total scores they get from different components are not the same among all subjects.

### Conclusions

To our knowledge, this was the first prospective study to assess the ability of diet quality indices to predict changes in lipid profiles in people of the Middle East countries. This research provides evidence that the HEI-2005 recommendations present a healthy eating pattern associated with reduced TG in an urban Iranian adult population. Considering the high levels of TG reported in Iran, the current results could be used in developing applicable guidelines to prevent hypertriglyceridaemia.

## ACKNOWLEDGEMENTS

This study was supported by grants from Research Institute for Endocrine Sciences, Shahid Beheshti University of Medical Sciences, Tehran, Iran. The authors would thank the participants and the TLGS personnel for their collaboration. We would like to thank Ms N. Shiva for English editing of the manuscript.

**Appendix 1.** Components and scoring criteria for the diet quality index-international

Component	Criterion	Point	Score range
Overall food group variety	≥1 serving(s) from each food group/d	3	0-15
Within-group variety from protein source	≥3 different sources/d	5	0-5
	2 different sources/d	3	
	From 1 source/d	1	
	None	0	
Total vegetables	≥100% recommendations^*^	5	0-5
	Otherwise	P	
Total fruits	≥100% recommendations^*^	5	0-5
	Otherwise	P	
Total grains	≥100% recommendations^*^	5	0-5
	Otherwise	P	
Protein	≥100% recommendations^†^	5	0-5
	Otherwise	P	
Calcium	≥100% recommendations^†^	5	0-5
	Otherwise	P	
Vitamin C	≥100% recommendations^†^	5	0-5
	Otherwise	P	
Iron	≥100% recommendations^†^	5	0-5
	Otherwise	P	
Fibre	≥100% recommendations^‡^	5	0-5
	Otherwise	P	
Total fat	≤30% energy/d	6	0-6
	>30–35% energy/d	3	
	>35% energy/d	0	
Saturated fat	≤7% energy/d	6	0-6
	>7–10% energy/d	3	
	>10% energy/d	0	
Cholesterol	≤300 mg/d	6	0-6
	>300–400 mg/d	3	
	>400 mg/d	0	
Sodium^¶^	<15^th^ percentile	6	0-6
	≥85^th^ percentile	0	
	Otherwise	P	
Empty calorie foods	≤3% energy/d	6	0-6
	>3–10% energy/d	3	
	>10% energy/d	0	
Macronutrient ratio (CHO:protein:fat)	55–65:10–15:15–30	6	0-6
	65–68:9–16:13–32	4	
	50–70:8–17:12–35	2	
	Otherwise	0	
Fatty acid ratio	PUFA/SFA=1–1·5; MUFA/SFA=1–1·5	4	0-4
	PUFA/SFA=0·8–1·7; MUFA/SFA=0·8–1·7	2	
	Otherwise	0	

^*^Based on 1,700, 2,200, and 2,700 energy levels introduced in Food Guide Pyramid 1992;

^†^According to dietary reference intake recommendations;

^‡^>20, 25, and 30 g for the 1,700, 2,200, and 2,700 energy levels introduced in Food Guide Pyramid 1992 respectively;

^¶^Based on the distribution of sodium content of foods consumed by the study subjects; CHO=Carbohydrate; d=Day; MUFA=Monounsaturated fatty acid; P=Proportionately; PUFA=Polyunsaturated fatty acid; SFA=Saturated fatty acid

**Appendix 2.** Components and scoring criteria for the healthy eating index-2005

Component	Criterion	Point	Score range
Total vegetables	≥1.1 cup equiv./1,000 kcal	5	0-5
	Otherwise	P	
Dark-green and orange vegetables and legumes	≥0.4 cup equiv./1,000 kcal	5	
	Otherwise	P	
Total fruits	≥0.8 cup equiv./1000 kcal	5	0-5
	Otherwise	P	
Whole fruit	≥0.4 cup equiv./1,000 kcal	5	0-5
	Otherwise	P	
Total grains	≥3.0 oz equiv./1,000 kcal	5	0-5
	Otherwise	P	
Whole grains	≥1.5 oz equiv./1,000 kcal	5	0-5
	Otherwise	P	
Diary	≥1.3 cup equiv./1,000 kcal	10	0-10
	Otherwise	P	
Meat and beans	≥2.5 oz equiv./1,000 kcal	10	0-10
	Otherwise	P	
Oils	≥12 grammes/1,000 kcal	10	0-10
	Otherwise	P	
Saturated fatty acid	≤7% energy/d	10	0-10
	≥15% energy/d	0	
	Otherwise	P	
Sodium^*^	1^st^ decile	10	0-10
	10^th^ decile	0	
	Otherwise	P	
SoFAAS	≤20% energy	20	0-20
	≥50% energy	0	
	Otherwise	P	

*Based on the distribution of sodium content of foods consumed by the study subjects; d=Day; P=Proportionately; SoFAAS=Total calories from solid fats and added sugars

**Appendix 3.** Components and scoring criteria for the Mediterranean dietary scale

Component	Criterion	Point	Score range
Vegetables	≥Median	1	0-1
	<Median	0	
Fruits	≥Median	1	0-1
	<Median	0	
Whole grains	≥Median	1	0-1
	<Median	0	
Refined grain	<Median	1	0-1
	≥Median	0	
Dairy products	<Median	1	0-1
	≥Median	0	
Red-to-white meat ratio	<Median	1	0-1
	≥Median	0	
Fish	≥Median	1	0-1
	<Median	0	
Nuts	≥Median	1	0-1
	<Median	0	
Legumes	≥Median	1	0-1
	<Median	0	
PUFA/SFA	≥Median	1	0-1
	<Median	0	

PUFA=Polyunsaturated fatty acid, SFA=Saturated fatty acid
